# Meta-analysis of genetic diversity and intercolony relatedness among reproductives in commercial honey bee populations

**DOI:** 10.3389/finsc.2023.1112898

**Published:** 2023-01-30

**Authors:** David R. Tarpy, Joel R. Caren, Deborah A. Delaney

**Affiliations:** ^1^ Department of Applied Ecology, Graduate Program in Biology—Evolution & Ecology, North Carolina State University, Raleigh, NC, United States; ^2^ USDA-ARS, Pollinator Health Center, Stoneville, MS, United States; ^3^ Department of Entomology & Wildlife Biology, University of Delaware, Newark, DE, United States

**Keywords:** Apis mellifera, breeding, relatedness, social insects, genetic structure

## Abstract

Honey bee colonies are large kin groups, each with a single mother queen and thousands of female workers. Queen bees are highly polyandrous, each mating with an average of approximately 12 drones from other colonies. We used a meta-analysis approach to compare the pedigree relationships of honey bee reproductives (queens and their mates) across five different studies and to quantify the overall genetic diversity of breeding populations. We compared the inferred genotypes of queens and their mates from microsatellite analyses of worker offspring from a feral Africanized honey bee population (which served as a negative control for inbreeding), an experimentally derived population of sister queens (which served as a positive control for inbreeding), and three separate commercially managed populations. We then compared the relatedness of all drones mated to each queen (mate-mate), all queens within each population (queen-queen), each queen with each of her mates (queen-mate), and all drones within each population (drone-drone). We found, as expected, the lowest levels of genetic similarity in the outcrossed population and highest levels of genetic similarity in the inbred population. Levels of genetic similarity among the managed honey bee populations were intermediate but closer to that of the inbred population. Genetic structuring of the entire breeding population resulted in two major subpopulations, likely deriving from breeders on the east and west coast. The effects that these findings have on the overall population genetic diversity of managed honey bees is discussed.

## 1 Introduction

The plight of honey bees and other pollinators has gripped the attention of scientists and the general public alike, bringing increased scrutiny of the many environmental stressors on bees, particularly in managed agricultural habitats (1–4). It is widely held that the three main challenges pollinators face are parasites and pathogens (5–7), pesticides and other environmental contaminants (8–10), and nutritional deficiencies particularly as a result of habitat loss (6, 11–13). In addition, these challenges interact in critical and highly complex ways. This conventional wisdom, however, inherently rests upon the underlying genetic architecture of pollinator populations, which is rarely accounted for in studies of bee health. Since phenotypes are a function of genetics, the environment, and their interaction, quantifying the genetic background and relatedness of bees is imperative to fully understanding how environmental stressors may affect them.

In managed honey bees, population genetic structure has been investigated at several levels. On the evolutionary timeframe, phylogenetic studies have determined aspects of speciation and adaptive radiation (14–16). This provides an important historical perspective since it demonstrates the genetic diversity at the species or subspecies level, but since most imported *Apis* subspecies were of the M and C lineages (17), diversity at the evolutionary level does not likely impact how managed colonies interact with their local environment. At a more proximate and ecological timescale, Harpur et al. (18) tested managed honey bee populations in the US and Europe, then compared them to wild populations in Africa, Eastern Europe, and Western Europe. They concluded that there is significantly more admixture and genetic diversity in the introduced range, which prompted a debate (19, 20) about the merits and disadvantages of panmixis in managed honey bee populations (17, 21, 22). There have also been several studies comparing managed and putatively feral US honey bee colonies (23–26), showing differences in their relative genetic diversity. Finally, determining the genetic diversity of breeding programs has been of particular interest to researchers (27–29) due to a relatively small number of queen producers (mainly in Hawaii, California, and the southeast) accounting for the majority of genetic stock among beekeepers in the US (30).

Genetic diversity at these higher levels is critical to that at the colony level. One of the leading evolutionary explanations for hyperpolyandry (extreme female multiple mating) in honey bees is that the resulting increased intracolony genetic diversity is adaptively favored in many ways (reviewed in (31–34)). As can be seen in examples of genetic bottlenecking in other agricultural or livestock systems (35–40), it is important to the long-term sustainability of a healthy honey bee population to maximize genetic diversity and to avoid genetic bottlenecks among generations over the long term (30, 41–43).

Because of our previous work analyzing the genetic relatedness of individuals within colonies in an effort to estimate the effective paternity frequencies of honey bee queens (see below), we have a unique opportunity to conduct a meta-analysis on the individual genotypes of queens and their mates to quantify the degrees to which honey bees are related at the colony, operation, and population levels. Our goals were to quantify the average relatedness among breeding individuals in different honey bee populations, compare the pedigree relationships among breeding individuals, and determine the genetic structure of the US managed honey bee population.

## 2 Materials and methods

### 2.1 Datasets

We have five published datasets of workers from separate honey bee colonies genotyped to estimate the effective paternity frequencies of their queen mothers. From these progeny genotypes, we were able to infer each queen’s genotype, as well as those of each of the drones that mated with her, at the same six highly polymorphic microsatellite loci (while each study used more than six molecular markers to ascertain worker genotype, this set of six microsatellites were common among them). This enabled us to compare the five populations’ relative genetic relatedness both within and among colonies and breeding populations (reviewed in **Table 1**).

**Table 1:** Summary of each dataset (including the number of queens and the average effective paternity frequency) and allelic richness per locus by experimental study, based on a minimum sample size of 14 diploid workers.

		Experimental study				
		AHB	Migratory	Breeding 1	Breeding 2	Experimental
No. colonies (queens)		17	79	22	61	33
Location		Rural Arizona	Eastern seaboard	National survey	National survey	Raleigh, NC
Workers per colony (± SEM)		43.5 ± 3.69	91.2 ± 12.31	116.5 ± 5.34	45.9 ± 1.97	47.0 ± 0.31
Effective paternity frequency (± SD)		20.0 ± 8.46	13.6 ± 6.76	16.0 ± 9.48	17.0 ± 8.98	14.2 ± 4.97
Reference		(44)	(45)	(46)	(47)	(48)
**Microsatellite locus**	A113	6.46	4.06	4.63	5.14	4.79
	A24	4.65	3.71	2.96	4.69	3.93
	A88	9.38	2.51	4.23	2.96	2.00
	Ap43	11.71	5.47	5.10	6.02	2.88
	Ap81	6.00	2.66	2.00	2.64	2.00
	B124	5.98	4.52	5.98	7.03	6.17
	Average	7.36a	3.82b	4.15b	4.75ab	3.63b
	Sum	44.19	22.93	24.89	28.48	21.77

Our first dataset was from Tarpy et al. (45), in which we tracked commercial migratory operations over the course of a year (‘Migratory’ population). In doing so, we sampled an average of 91.2 ± 12.31 workers from each of 79 colonies at the beginning of the beekeeping season, genotyped them to infer queen and drone marker sets, and determined how intracolony genetic diversity was associated with queen and colony survival (45). Our second dataset was from Delaney et al. (46), where we purchased commercially produced queens from across the nation, then quantified their physical, insemination, and mating quality (‘Breeding 1’ population). This involved analyzing another 22 colonies by genotyping worker offspring to infer queen and drone marker sets. Our third dataset was from Tarpy et al. (47), which was a separate and larger assessment of the reproductive quality of commercially produced queens (n=61) and their mates (‘Breeding 2’ population).

Our final two datasets served as comparative controls to the commercial populations. First, we analyzed the feral population sampled in the desert southwest (44), capturing workers from 17 unmanaged Africanized colonies (‘AHB’ population). This dataset served as a “positive control,” since we would expect maximal outcrossing and minimal genetic relatedness among the queens and drones in this non-managed population. Second, we analyzed 33 colonies from Tarpy et al. (48) that were part of a highly controlled experimental design of empirically produced high- and low-quality queens (‘Experimental’ population). In this study, all queens were grafted from the same open-mated mother queen, and as such this dataset served as a “negative control.” Thus, although we could not control the genetic diversity of the parental drones, we would expect minimal genetic diversity among the sister queens in this population.

### 2.2 Pedigree analysis

**Figure 1:**
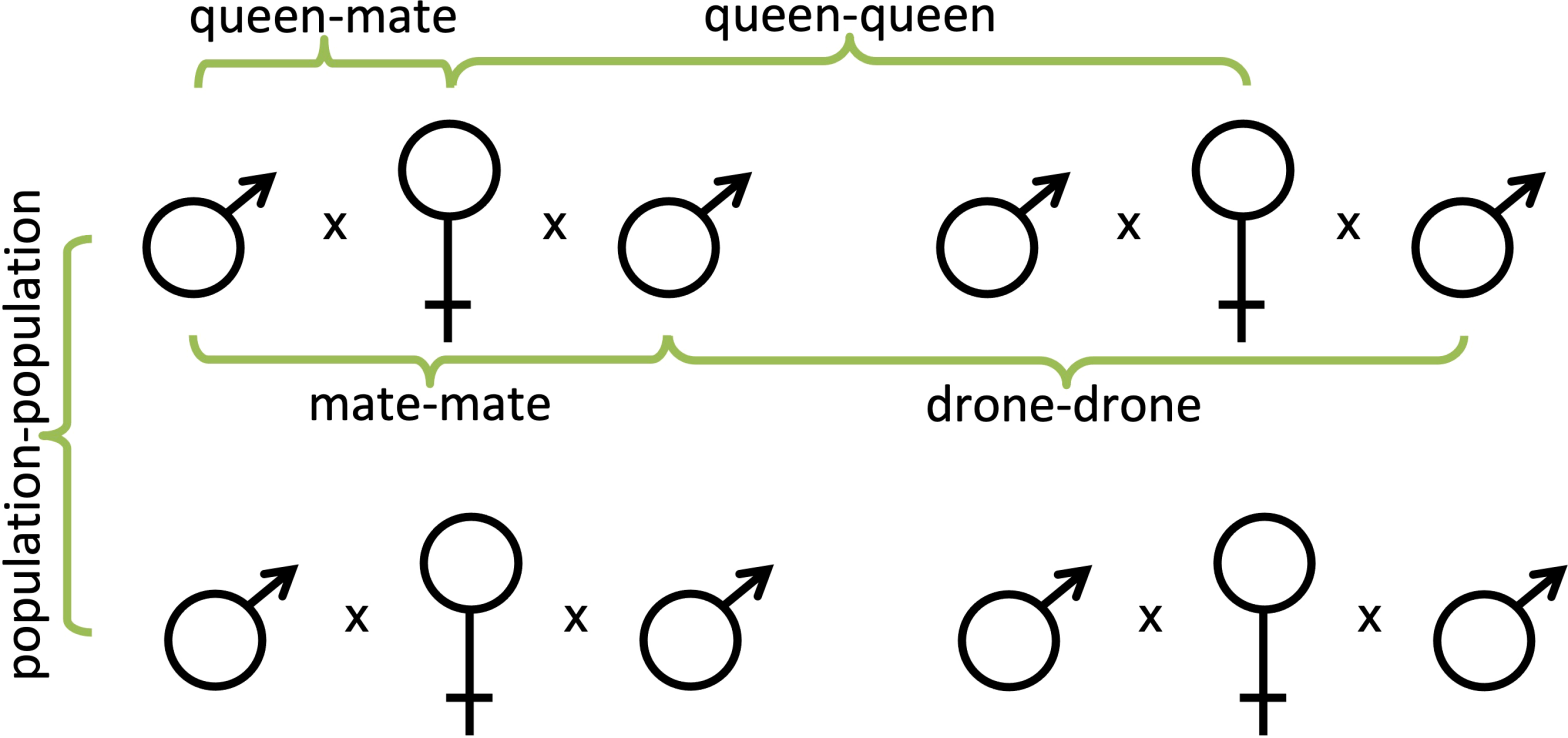
Pedigree diagrams of all of the pairwise comparisons made within and among honey bee colonies; queen-mate, mate-mate, queen-queen, and drone-drone.

Knowing all of the genotypes of the queens and of the drones with which they mated in all five studies, we were able to compare the relatedness among them. In doing so, we were able to determine the average genetic relatedness between each queen and her mates (queen-mate), between mates of the same queen (mate-mate), between the queens within each population (queen-queen), and between drones mating with different queens within the same population (drone-drone; **Figure 1**). We used RELATEDNESS^®^ (v. 5.0.8) (49) to estimate each pairwise comparison within each population for a total ~8.5 million individual calculations. Computer code was then written to compare to known relatedness values that were not significantly different from known pedigree relationships (e.g., siblings, cousins). This enabled us to not only quantify the average relatedness among the different pairings of individuals but also the proportion of individuals that were related by common decent (or at least not statistically distinguishable from the same genetic pedigree).

### 2.3 Population analyses

The population genetics software GENETIX™ was used to calculate allele frequencies. Hardy-Weinberg equilibrium, linkage disequilibrium, heterozygote deficiency, heterozygosity (observed and expected), genic and genotypic differentiation, and population pairwise *F_st_* values were estimated using the software program *FSTAT* (50). Using the software HP-RARE (51), average number of alleles per locus (*n_a_*), allelic richness (*a_g_*), and expected heterozygosity (*H_e_*) were calculated for each feral population. Rarefaction takes into account any differences in sample sizes (52). The *Bottleneck* (53) program was also used to detect reductions in effective sizes in populations by determining excessive heterozygosity using three kinds of statistical tests: sign test, standardized differences test, and the Wilcoxon’s signed rank test. The program STRUCTURE (54) was used to evaluate genetic structure among all populations. This program produces likely clusters or distinct groups using the allele frequencies of neutral markers in a Bayesian model-based clustering method and can be used to detect sub-structure within a population. Three runs for each value of K from K = 1 - 6 were run, with 50,000 burn-ins and 100,000 replications after burn in.

## 3 Results

**Figure 2:**
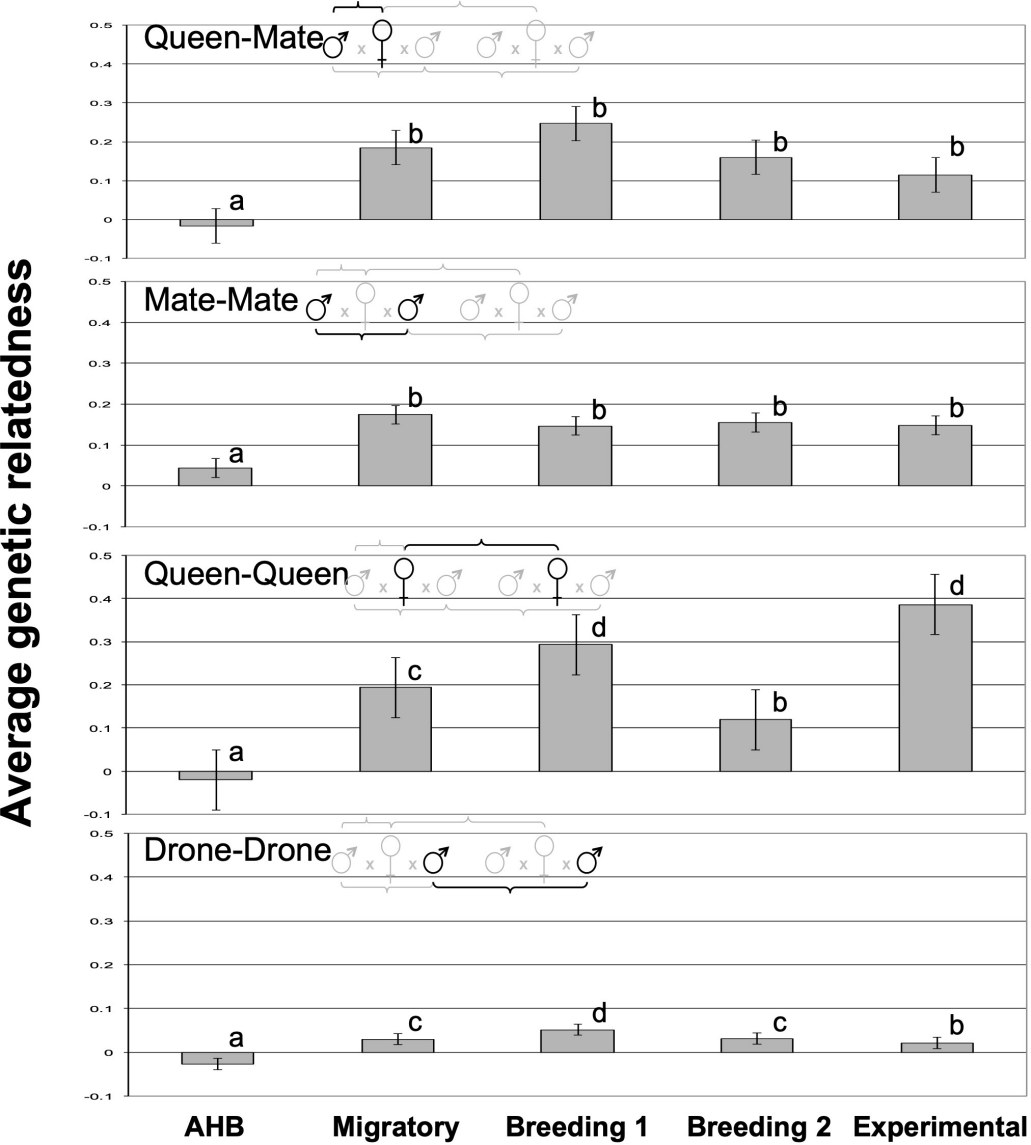
The average genetic relatedness of each pairwise comparison of queens and their mates among the five experimental groups. All letters indicate significantly different means according to Tukey post-hoc tests (p<0.05).

We found significant differences in the average relatedness among the five breeding populations for all queen- and drone comparisons (**Figure 2**). As expected, the average relatedness of queens to their mates (queen-mate) was not significantly different from zero for those in the AHB population (-0.035 ± 0.035), but those of all managed populations were significantly higher and not significantly different from each other (*F*_4,207 =_ 7.06, *p*<0.0001). Similarly, the AHB population had the lowest mate-mate relatedness (albeit significantly higher than zero; 0.049 ± 0.015) with all other populations being significantly higher but again not statistically different from each other (*F*_4,207 =_ 8.94, *p*<0.0001). The relatedness among queens within each population (queen-queen) followed a different pattern, with values in the two “control” populations as expected; queen relatedness in the AHB population were not significantly different from zero (-0.040 ± 0.033), and queens in the Experimental population had the highest relatedness (since they were known sisters; 0.361 ± 0.028). The three commercial populations were numerically intermediate and all above zero (*F*_4,207 =_ 64.4, *p*<0.0001), with the Breeding 1 population being statistically equivalent to the Experimental population, the Migratory population being significantly lower, and the Breeding 2 population being between the Migratory and AHB populations (**Figure 2**).

Differences in average relatedness among drones mating with different queens within the same population demonstrated some particularly intriguing patterns (**Figure 2**). Again, the drones in the AHB population were maximally outbred and had an average relatedness below zero (-0.032 ± 0.006). All other populations had average relatedness values above zero, with the Migratory and Breeding 2 population being statistically equivalent and significantly lower than the Breeding 1 population (*F*_4,207 =_ 51.9, *p*<0.0001). Interestingly, the Experimental population drone-drone relatedness was statistically lower than that of all commercial populations (0.016 ± 0.008), albeit still higher than the AHB population, suggesting that our experimental research apiary has a higher genetic diversity of drones than do the tested commercial populations.

**Figure 3:**
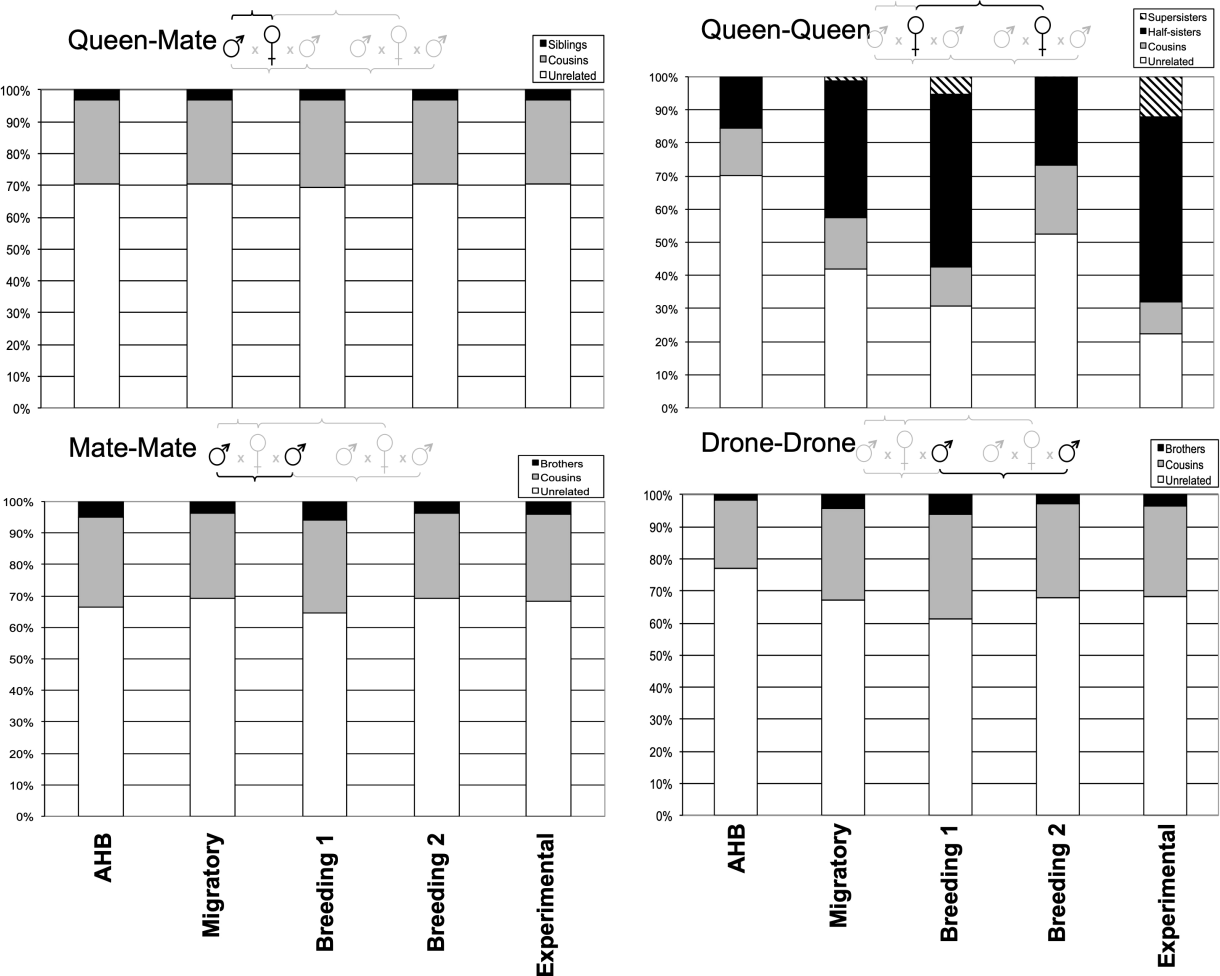
Relative proportions of unrelated and related individuals for each pairwise comparison of queens and their mates among the five experimental groups.

Alternatively, when we estimated the key Mendelian relationships among the reproductives within and among colonies across the five populations (**Figure 3**), we only found significant differences in the queen-queen comparisons (*χ^2^*_12_ *^=^* 94.6, *p*<0.0001) and none in the queen-mate (*χ^2^*_8_ *^=^* 0.01, *p*=1.00), mate-mate (*χ^2^*_8_ *^=^* 0.001, *p*=1.00), or drone-drone comparisons (*χ^2^*_8_ *^=^* 7.49, *p*<0.48). For queens, our AHB and Experimental populations were minimally and maximally inbred, respectively, as expected. The three commercial populations, however, were all intermediate, each with >48% of the queens being the genetic equivalent of supersisters (*G*=0.75), half-sisters (*G*=0.25), or cousins (*G*=0.125) (**Figure 3**).

There were no differences in allelic richness among the six microsatellite loci (**Table 1**), with all loci having 3.63-7.36 alleles in each study population (*F*_5,24 =_ 1.80, *p*=0.15). There were, however, significant differences in allelic richness across the studies (*F*_4,25 =_ 4.35, *p*<0.001), with the AHB population having significantly more alleles than the Breeding 1, Migratory, and Experimental groups with the Breeding 2 population being intermediate. The multilocus *F_st_* values for all pairwise comparisons were highly significant (**Table 2**).

**Table 2:** *F_st_* values for all pairwise comparisons of the five experimental studies.

	AHB	Migratory	Breeding 1	Breeding 2	Experimental
AHB	0				
Migratory	0.0807*	0			
Breeding 1	0.1055*	0.0488*	0		
Breeding 2	0.0785*	0.0143*	0.0324*	0	
Experimental	0.1634*	0.1353*	0.1656*	0.1157*	0

P-values obtained after 200 permutations. * = p<0.005.

**Figure 4:**
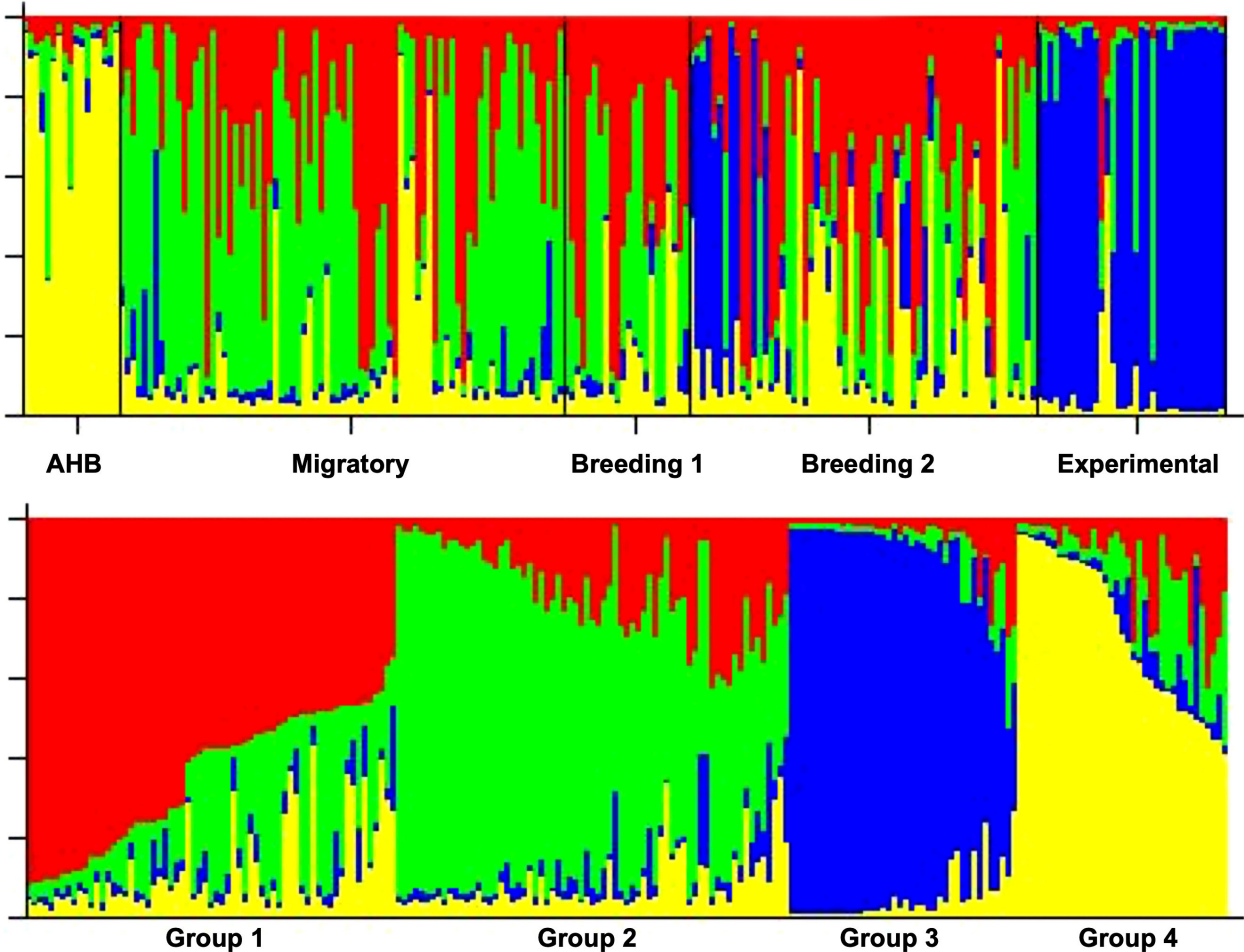
Top: STRUCTURE diagrams of the individuals (queens) from all 5 data sets (populations) demonstrating assignment into different genetic clusters (*K*). Bottom: K = 4 based on Ln P(D) and ΔK.

The five experimental groups formed distinct clusters when analyzed with STRUCTURE (**Figure 4**). When all data were analyzed together, the optimal number of distinct genetic populations was K = 4 based on (Ln P(D)) (54) and (Δ K) (55). The maximal value of Ln P(D) = -3718.5 for an L(K) of 6 based on six microsatellites from 211 queens. Groups 3 and 4 derive from the distinct AHB and Experimental populations, respectively, suggesting that there are two breeding populations in the three studies of managed stock (**Figure 4**).

## 4 Discussion

The results of our meta-analysis suggest that commercial breeding populations of managed honey bees are more inbred than maximally outcrossed but that there is sufficient and significant genetic variation depending on the level of analysis. While the five experimental datasets do not represent unique or separate breeding populations (i.e., some were surveys of multiple breeders whereas others were specific to certain operations) and thus do not represent a systematic sampling of all potential breeding stock in the US, they still represent many of the relatively small number of queen-producing operations and thus most managed honey bees. Since a small number of queen producers provide the majority of queens purchased by beekeepers each year (27, 30), our comparisons are valid representative examples of all potential breeding populations.

Our relatedness and pedigree analyses suggest that queens and drones within breeding populations can often be related by descent, thus demonstrating the potential for inbreeding. However, it appears that the overall effect on the average genetic relatedness within colonies is minimal. Perhaps most important is when queens mate with brothers or those closely related by descent, since this has the highest potential for resulting in homozygosity at the sex locus and other deleterious genetic combinations. We found that queen-mate relatedness among the five studies was significantly lower in the AHB feral population (as expected) compared to all others, suggesting that managed honey bees are not as outcrossed as they could be. However, all five groups had nearly identical queen-mate pedigrees, where ~70% of the queens mated with drones that were statistically unrelated (and less than 5% were genetically indistinct from brothers) irrespective of their mating population, including the unmanaged Africanized colonies. Another comparison of interest is the queen-queen analysis, which determines the likelihood of two queens being related by descent. This shows clear and intentional differences between the minimally inbred AHB and maximally inbred Experimental group. The three commercial groups were intermediate but much closer to having a higher likelihood of supersisters, half-sisters, or cousins. This finding is not surprising, given the standard methods of commercial queen rearing (56); while a given operation may use multiple breeder queens from which to graft young larvae to produce daughter queens, those from any given cohort likely derive from the same breeder queen and thus have higher average relatedness.

When looking at the population genetics of all queens, our results suggest that there is minimal structure within commercial honey bees, highlighting that the population in the US is not completely panmictic but nearly so (18). While accounting for the known AHB and Experimental populations, there appears to be only two distinct genetic populations across all queens from commercial managed stock. While our data do not permit any geographic inferences to be drawn, it is possible that this represents some genetic distinctions between queens reared in the southeast and west, where many if not most large-scale commercial queen producers are clustered. It would be interesting to conduct some further and systematic sampling and analysis of these two major queen-producing regions to quantify the degree of genetic overlap. Moreover, our results suggest that there is fairly clear, if limited, genetic introgression of AHB alleles into commercial stock (57–60).

This meta-analysis demonstrates that investigations into genetic diversity in honey bees can benefit from multiple perspectives. While population genetics can reveal overall trends and quantify the degree of potential diversity, Mendelian relationships are more critical at capturing functional bottlenecks that manifest at the colony level. Priority should be given to assessing genetic diversity within individual breeding populations at each of these levels to minimize inbreeding and hence optimize sustainability of the breeding stock of managed honey bees. In doing so, breeding programs should regularly test for allelic diversity (especially at the *csd* locus) to avoid inbreeding depression. Moreover, breeding programs with the goal of local environmental adaptation should eliminate or minimize admixture of genetic stock by maintaining large, closed breeding populations so that selection can manifest.

## Data availability statement

The data analyzed in this study is subject to the following licenses/restrictions: Previously published material. Requests to access these datasets should be directed to David Tarpy, drtarpy@ncsu.edu.

## Author contributions

All authors equally contributed to the experimental design and final writing of the manuscript. JC was responsible for developing the RELATEDNESS simulations and pedigree comparisons, as well as sample processing and genotype generation in the original studies. DD was involved in sample collection, data processing, and population-genetic analyses, with DT supporting these efforts as well as writing the initial draft, statistical analyses, and figure creation. DT and DD were responsible for acquiring funding. All authors contributed to the article and approved the submitted version.
